# The usage of clozapine in a patient with schizophrenia and epilepsy: A case report

**DOI:** 10.1192/j.eurpsy.2021.2136

**Published:** 2021-08-13

**Authors:** M. Karnickas, G. Bulotaite, V. Rutkauskas

**Affiliations:** 1 Institute Of Clinical Medicine, Clinic Of Psychiatry, Vilnius University Faculty of Medicine, Vilnius, Lithuania; 2 Faculty Of Medicine, Vilnius University, Vilnius, Lithuania; 3 Institute Of Health Sciences, Department Of Public Health, Vilnius University Faculty of Medicine, Vilnius, Lithuania

**Keywords:** clozapine, Epilepsy, treatment resistant schizophrenia, seizures

## Abstract

**Introduction:**

The usage of clozapine in patients with schizophrenia and epilepsy raises many challenges due to the epileptogenic effects of the drug. There is little data about using clozapine for treatment of treatment-resistant schizophrenia (TRS) accompanied by epilepsy.

**Objectives:**

To present a case report illustrating a patient diagnosed with epilepsy and TRS, successfully treated with clozapine.

**Methods:**

A literature review on “PubMed” database was conducted, using the keywords: clozapine, antipsychotics, epilepsy, seizures, psychoses. Information regarding the clinical case was obtained by consulting the patient’s file.

**Results:**

We present a woman, 33 years old with mild intellectual disability and a 10-year history of schizophrenia. At the age of 24, the patient lost consciousness and experienced tonic – clonic seizure, but epilepsy was not diagnosed. The patient was hospitalized multiple times due to positive symptoms of schizophrenia and suicidal thoughts. Various combinations of neuroleptics and electroconvulsive therapy were used for treatment without therapeutic effect. At the age of 32, a diagnosis of TRS was established, leading to treatment with a moderate clozapine dose (400mg/day). At the fifth month of treatment with clozapine un episode of generalized seizures occurred, leading to a diagnosis of idiopathic generalized epilepsy. Since then, the patient was treated with levetiracetam and the dosage of clozapine was lowered to 275mg/d. The seizures did not re-occur and clinical response to the psychiatric treatment was positive.

**Conclusions:**

This case report highlights how clozapine can be used safely and effectively for patients with TRS and epilepsy and how the epileptogenic effects can be moderated by using antiepileptics in the overall treatment scheme.

**Disclosure:**

No significant relationships.

